# Modulation of lignin and anthocyanin homeostasis by *GTP cyclohydrolase1* in maize

**DOI:** 10.1111/pbi.70061

**Published:** 2025-03-28

**Authors:** Mingyue Zhang, Xiaohan Li, Xiao Wang, Shuzhen Jiang, Junli Zhang, Mingfei Sun, Zixian Zhou, Jinxiao Zhang, Mengyao Li, Yanxiao Lv, Enlong Qi, Ziang Tian, Hongjie Zhu, Xuebin Zhang, Xiangyu Zhao, Changcheng Xu, Thomas Lübberstedt, Xiansheng Zhang, Xuerong Yang, Chao Zhou, Hongjun Liu

**Affiliations:** ^1^ State Key Laboratory of Crop Biology, College of Life Sciences Shandong Agricultural University Tai'an Shandong China; ^2^ State Key Laboratory of Crop Stress Adaptation and Improvement, Henan Joint International Laboratory for Crop Multi‐Omics Research, School of Life Sciences Henan University Kaifeng China; ^3^ Key Laboratory of Efficient Utilization of Non‐Grain Feed Resources College of Animal Science and Technology, Shandong Agricultural University Tai'an Shandong China; ^4^ Biology Department Brookhaven National Laboratory Upton NY USA; ^5^ Department of Agronomy Iowa State University Ames IA USA; ^6^ Yazhouwan National Laboratory Sanya Hainan China

**Keywords:** maize, lignin, anthocyanin, GTP Cyclohydrolase1 (GCH1), brown midrib

## Abstract

Maize is a key biomass resource with wide agricultural applications. Anthocyanins, potent antioxidants, offer health benefits like reducing oxidative stress. The biosynthesis of anthocyanins competes with that of lignin for shared metabolic precursors, which can lead to trade‐offs in plant growth and feed quality. Higher lignin content can decrease silage digestibility, posing challenges for livestock feed. The maize *brown midrib 6* (*bm6*) mutant, known for reduced lignin, has an unclear genetic basis. Here, we identify *ZmGCH1* as the candidate gene for *bm6* through fine mapping. Mutations in *ZmGCH1* shift precursors from lignin to anthocyanin biosynthesis. Furthermore, we show that ZmGCH1 interacts with ZmPEBP15 to modulate chalcone synthase activity, thereby stabilizing the allocation of precursors between lignin and anthocyanin pathways. To evaluate the practical implications of our findings, we introduced the *bm6* mutation into Zhengdan958 and Xianyu335. In vitro rumen digestion assays confirmed that the introduction of the *bm6* mutation significantly improved silage digestibility. This discovery not only holds great potential for enhancing silage digestibility but also provides a broader strategy for optimizing maize production to better meet the increasing demands of both the food and livestock feed.

## Introduction

The maize *brown midrib* (*bm*) mutant, first identified in 1931 (Jorgenson, 1931), is a classic lignin mutant. Among the six reported mutants (*bm1* to *bm6*), *bm3* was the first cloned and shown to encode caffeate‐O‐methyltransferase (COMT), a key enzyme in lignin biosynthesis (Vignols *et al*., 1995). Similarly, *BM1* encodes cinnamyl alcohol dehydrogenase (CAD), critical for lignin synthesis, with mutants showing reduced lignin content, particularly in G and S monomers (Halpin *et al*., 1998). The *BM2* and *BM4* genes were later cloned, revealing roles in one‐carbon metabolism: *BM2* encodes methylene tetrahydrofolate reductase (MTHFR) (Tang *et al*., 2014), and *BM4* encodes folylpolyglutamate synthetase (FPGS) (Li *et al*., 2015), both regulating S‐adenosylmethionine (SAM) synthesis, which is a methyl donor essential for lignin biosynthesis (Cossins and Chen, 1997; Li *et al*., 2015; Mehrshahi *et al*., 2010). The *bm5* mutant encodes 4‐coumarate‐CoA ligase (Zm4CL1), another key enzyme in lignin biosynthesis (Boerjan *et al*., 2003; Lavhale *et al*., 2018), with mutations altering monomer composition but not total lignin content (Ali *et al*., 2010; Xiong *et al*., 2019).

The *BM6* gene was initially identified as a novel maize *brown midrib* (*bm*) gene through the efforts of our research collaborator (Chen *et al*., 2012). In the years following this discovery, we conducted extensive fine mapping of the region surrounding the *BM6* locus and confirmed that a mutation in the *ZmGCH1* gene (*GTP cyclohydrolase 1*) is responsible for the *bm6* phenotype. The mutation involves a 141‐bp Mu insertion located in the second intron of ZmGCH1, situated 181 bp downstream of the exon–intron junction. Notably, this region aligns precisely with the location mapped by Leonard *et al*. (2022), further corroborating our findings. *ZmGCH1* plays a crucial role in the first step of tetrahydrofolate biosynthesis, a key cofactor for one‐carbon unit transfers such as methylation (Cossins and Chen, 1997). Additionally, *ZmGCH1* functions upstream of *BM4*, co‐regulating lignin biosynthesis pathways (Hanson and Roje, 2001; Leonard *et al*., 2022).

Most of the bm mutants discovered so far show a significant reduction in lignin content. Lignin biosynthesis originates in the phenylalanine metabolic pathway and shares the common precursor *p*‐coumaroyl‐CoA with anthocyanin synthesis (Fraser and Chapple, 2011). In the phenylpropanoid pathway, *p*‐coumaroyl‐CoA serves as a substrate for both chalcone synthase (CHS) and hydroxycinnamoyl‐CoA shikimate/quinate hydroxycinnamoyl transferase (HCT). HCT catalyses the synthesis of shikimate and quinate esters of coumaric acid, which are intermediates in the production of guaiacyl and syringyl lignin units (Franke *et al*., 2002; Schoch *et al*., 2001). In *Arabidopsis thaliana*, silencing the HCT gene disrupts lignin biosynthesis, leading to reduced syringyl lignin and elevated levels of flavonoids, including flavonols and anthocyanins. This metabolic shift occurs as lignin synthesis is impaired, redirecting flux toward flavonoid production through increased CHS activity (Hoffmann *et al*., 2004).

Chalcone synthase (CHS) is a key enzyme in flavonoid biosynthesis, catalysing the condensation of *p*‐coumaroyl‐CoA with three malonyl‐CoA molecules to produce naringenin chalcone. This unstable intermediate is rapidly converted by chalcone isomerase (CHI) into naringenin, a stable precursor for diverse flavonoids, including flavonols, flavones and anthocyanins. These compounds play critical roles in plant pigmentation, UV protection and defense against pathogens. The downstream biosynthesis of specific flavonoids involves specialized enzymes, such as flavonoid 3′,5′‐hydroxylase (F3′5′H), flavone synthases (FS1/2), leucoanthocyanidin reductase (LAR) and UDP‐glucose flavonoid 3‐O‐glucosyltransferase (UFGT) (Deng *et al*., 2014).

Flavone synthase II (FNS II) catalyses the conversion of naringenin into tricin, a flavonoid that serves as a unique lignin monomer beyond the traditional cinnamic acid pathway (Lam *et al*., 2017). Tricin contributes to lignin polymer formation by coupling with both non‐acylated and C‐acylated monomers through free radical oxidation (del Río *et al*., 2012, 2020; Lam *et al*., 2021). In rice, mutants deficient in *OsFNSII* (*CYP93G1*) show reduced tricin content in lignin (Lam *et al*., 2015), resulting in altered cell wall structure, reduced total lignin, a changed S/G lignin ratio and improved cell wall digestibility (del Río *et al*., 2012). In addition, Naringenin produces Naringin under the action of glycosyltransferase (Bowles *et al*., 2006; Holton and Cornish, 1995; Jez *et al*., 2002).

In this study, we identified a mutation in the *ZmGCH1* gene as the cause of the brown midrib phenotype in *bm6* mutants. This mutation significantly increased anthocyanin accumulation in *bm6_*NIL lines. RNA‐seq, metabolic profiling and biochemical analyses revealed that the *ZmGCH1* mutation disrupts tetrahydrofolate metabolism and alters the metabolic flux of *p*‐coumaroyl‐CoA, redirecting it from lignin to the flavonoid biosynthesis pathway. This shift in metabolic flux is likely modulated by the interaction between ZmGCH1 and ZmPEBP15, which appears to influence CHS activity. To evaluate its agricultural potential, the *bm6* mutation was introduced into two elite maize varieties, Zhengdan 958 and Xianyu 335. In vitro rumen digestion experiments demonstrated improved silage digestibility, highlighting *bm6* maize as a promising strategy for enhancing livestock energy intake, addressing nutritional challenges and promoting sustainable agricultural practices.

## Results

### Map‐based cloning of gene responsible for the *bm6* mutant

**Figure 1:**
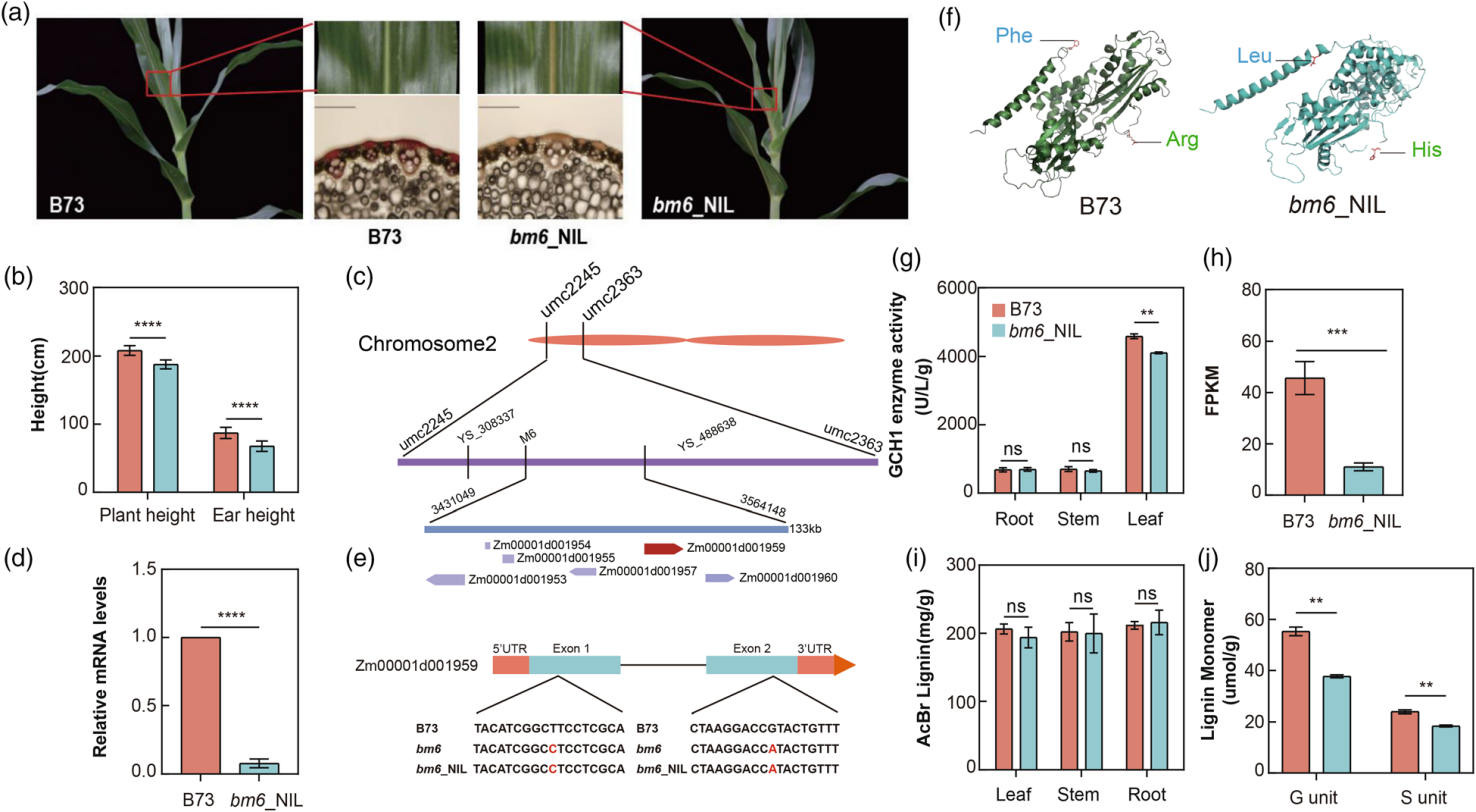
Map‐based cloning of gene responsible for the *bm6* mutant. (a) Leaf midvein phenotypes and results of phloroglucinol staining of *bm6*_NIL and B73. (b) Plant height and ear height of B73 and *bm6*_NIL during the ripening period. Error bars represent the ±standard deviation of three biological replicates (****P < 0.0001). (c) *bm6* fine localization was performed using BC_8_F_2_ population constructed by *bm6* and B73. (d) Expression levels of *ZmGCH1* gene in B73 and *bm6*_NIL leaves by quantitative polymerase chain reaction (qPCR). The mRNA level was normalized to that of *Actin*. Error bars represent the ± standard deviation of three biological replicates (****P < 0.0001). (e) Gene structure of Zm00001d001959, red bases represent SNP mutation sites. (f) AlphaFold 3 predicted ZmGCH1 protein structure in B73 and *bm6*_NIL. (https://golgi.sandbox.google.com/). (g) The ZmGCH1 enzyme activity of B73 and *bm6*_NIL at V8 stage was determined by spectrophotometry. Error bars represent the ± standard deviation of three biological replicates (**P < 0.01, ns *P* > 0.05). (h) Comparison of FPKM values of *Zm0001d001959* gene in the B73 and *bm6*_NIL transcriptome. (i) The lignin content of B73 and *bm6*_NIL at V8 stage was determined by spectrophotometry. Error bars represent the ± standard deviation of three biological replicates (ns *P* > 0.05). (j) The lignin monomer content of B73 and *bm*6_NIL during the ripening period. Error bars represent the ± standard deviation of three biological replicates (**P < 0.01).

To purify the genetic background for subsequent experimental analysis, we crossed the *bm*6 mutants with the commonly used inbred line B73 to establish a stable BC_6_F_7_ near‐isogenic line (NIL), *bm6*_NIL. The *bm6*_NIL exhibited the characteristic brown midrib phenotype consistent with previously reported *bm* mutants (Figure 1a), while its stem showed no colour difference compared to B73 (Figure S1a). Additionally, *bm6*_NIL displayed significantly reduced plant height, ear height and exhibited a dwarfed plant phenotype (Figure 1b and Figure S1b).

The *bm6* gene was previously mapped to a 180 kb interval on the short arm of chromosome 2 (Chen *et al*., 2012). Based on this, we screened BC_8_F_2_ populations derived from *bm*6 and B73 using molecular markers. Through comparative sequencing analysis with *bm6*, we successfully delimited the target gene region to 133 kb and identified six candidate genes within this region (Figure 1c and Figure S2a–e). The gene *Zm00001d001953* and *Zm00001d001959* exhibits a broad expression profile across various tissue types (Figure S3a–f). Apart from two tRNAs (*Zm00001d001954*, *Zm00001d001955*), expression differences in the remaining four genes between B73 and *bm6*_NIL were examined. Based on qRT‐PCR results, only *Zm00001d001959* showed significantly reduced expression among these genes (Figure 1d and Figure S4a–c). This gene encodes ZmGCH1, which functions upstream in the tetrahydrofolate (THF) synthesis pathway, with THF serving as a substrate for the FPGS enzyme encoded by the *bm4* gene (Bekaert *et al*., 2008). Taken together, *Zm00001d001959* was identified as a potential candidate gene responsible for the *bm6* mutants.

Subsequent sequencing of the candidate gene in B73 and *bm6*_NIL identified point mutations T > C and G > A in the coding regions (Figure 1e). These mutations led to amino acid substitutions: phenylalanine to leucine at position 37 and arginine to histidine at position 251. Such changes are expected to induce structural variations in the ZmGCH1 protein (Figure 1f). To further investigate the effects of the two amino acid mutations on protein function, we measured ZmGCH1 enzyme activity in the roots, stems, and leaves of B73 and *bm6*_NIL plants. A reduction in ZmGCH1 enzyme activity was detected only in the leaves, with no significant differences observed in other tissues (Figure 1g). These results suggest that the impact of the mutations on enzyme activity may be tissue‐specific. RNA‐seq analysis of *bm6*_NIL and B73 revealed that *ZmGCH1* was the only one among the six candidate genes to be significantly down‐regulated (Figure 1h, Table S1), further confirming *ZmGCH1* as the candidate gene for *bm6* mutants.

### Phylogenetic analysis and expression pattern of ZmGCH1

Our analysis revealed that ZmGCH1 contains two GTP cyclohydrolase domains using SMART (Simple Modular Architecture Research Tool) (Figure S5a). qRT‐PCR analysis indicated that *ZmGCH1* is expressed in roots, stems, leaves, midribs, tassel, ears and seeds (Figure S5b). Phylogenetic analysis of *ZmGCH1* and its homologues in various plants, along with amino acid sequence comparison of its first cyclohydrolase domain, confirmed its conservation in monocots and dicots (Figure S5c).

To ascertain the sub‐cellular localization of ZmGCH1, we fused the full‐length CDS of ZmGCH1 with the N‐terminal coding sequence of enhanced green fluorescent protein (eGFP), employing free eGFP as a control for the transient assay. The result showed that GFP signals could be detected in the cytoplasm, indicating that ZmGCH1 is localized in the cytoplasm (Figure S6a). To further verify the subcellular localization of ZmGCH1, the red fluorescent protein mCherry, which localizes in the cytoplasm and nucleus, and ZmGCH1‐eGFP were transiently co‐expressed in maize protoplasts. Free eGFP co‐transformed with mCherry served as a negative control. The results demonstrated that the green fluorescence from the ZmGCH1‐eGFP coincided with the cytoplasmic red fluorescence from mCherry, confirming that ZmGCH1 is localized in the cytoplasm (Figure 4c). Furthermore, through co‐localization analysis with AtCBL, we have excluded the potential for GCH1 to localize at the cell membrane (Figure S7b).

### The lignin content in *bm6*_NIL is decreased

We investigated the qualitative differences in lignin deposition in the midrib, root and stem tissues between *bm6*_NIL and B73 using phloroglucinol‐HCl staining. Significant differences were observed only in the midrib, where *bm6*_NIL exhibited a marked reduction in lignin deposition compared to B73 (Figure 1a). No significant differences were detected in the roots and stems (Figure S1c), aligning with the observed plant phenotypes.

To further evaluate lignin levels in *bm6*_NIL mutants, we measured the AcBr lignin content in the leaves, stems and roots of B73 and *bm6*_NIL at the V8 stage using ultraviolet spectrophotometry (Figure 1i). The AcBr lignin content in *bm6*_NIL leaves was reduced, though not significantly. Despite the total lignin content in *bm6*_NIL mutants not showing a significant decrease, there was a more pronounced reduction in lignin monomers (Figure 1j). This finding is similar to other brown midrib mutants such as *bm*2 and *bm*5 (Barrière *et al*., 2004; Marita *et al*., 2003; Méchin *et al*., 2014; Sattler *et al*., 2010; Vermerris and Boon, 2001; Xiong *et al*., 2019). The absence of a significant reduction in total lignin levels may be attributed to the complexity of lignin biosynthesis, with an increase in tricin potentially compensating for the decreased levels of G and S lignin monomers (Tables S4 and S6).

To assess whether changes in total lignin content affect the morphology of various tissues or cells, paraffin sections of the midrib, stem and root from B73 and *bm6*_NIL at the V8 stage were prepared for histological analysis (Figure S4d). The results showed no significant morphological differences between the *bm6*_NIL mutants and B73 in the epidermal cells, vascular bundles, vascular bundle sheath, sclerenchyma, xylem and phloem of the leaf midrib, indicating that changes in lignin content do not significantly impact the tissue structure at the leaf midrib. Furthermore, the number and structure of vascular bundles in the stem, as well as the vascular cylinder structure in the root, were also unchanged in the *bm6*_NIL mutants.

### Transcriptomic analysis to identify genes associated with the brown midrib phenotype in the *bm6*_NIL

To investigate the mechanism of lignin biosynthesis in *bm6* mutants and evaluate the effect of the mutation on gene transcription levels, RNA was extracted from the leaves of B73 and *bm6*_NIL plants at the V8 stage. The RNA samples were then analysed via RNA sequencing to determine transcriptomic changes.

**Figure 2:**
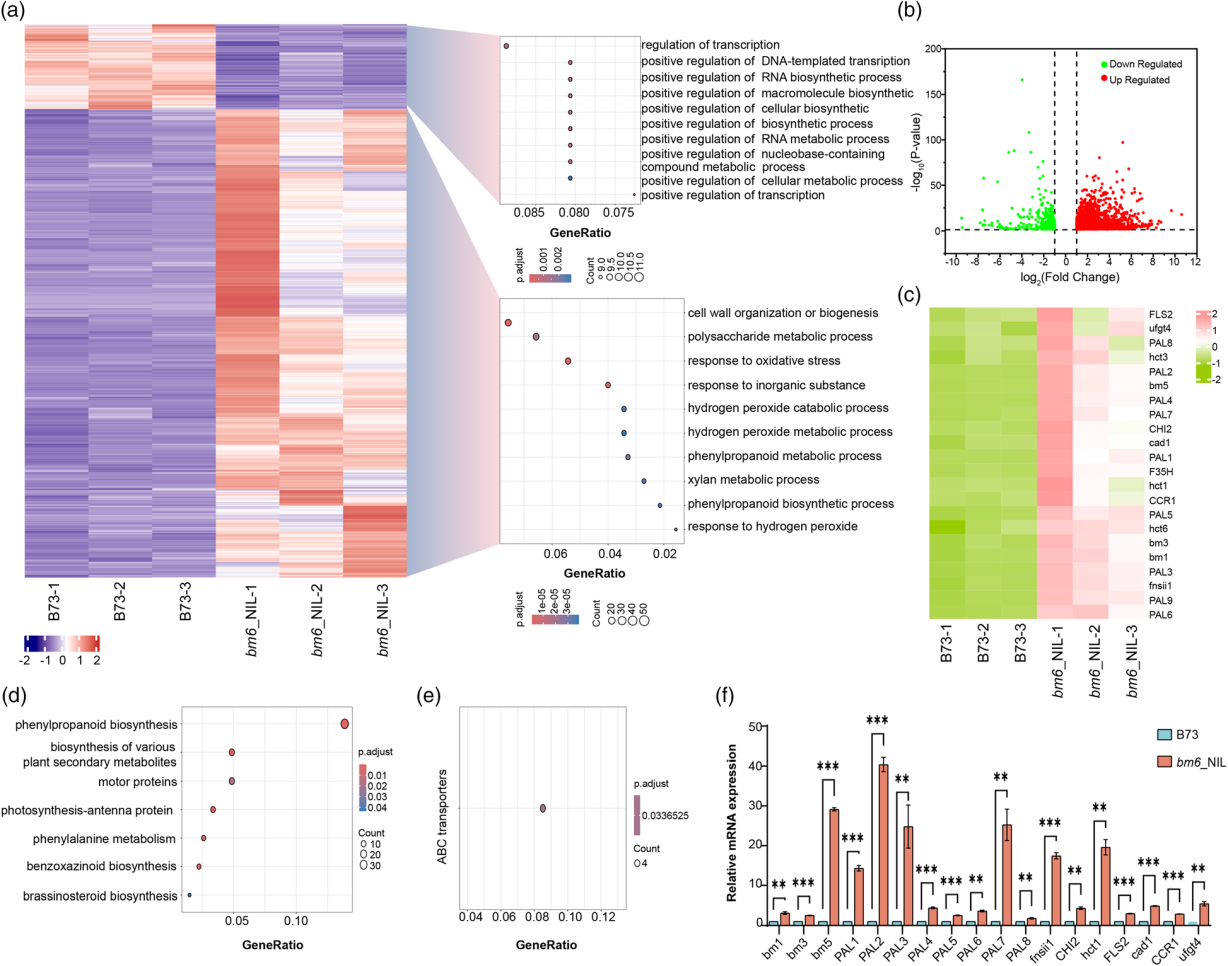
Transcriptomic analysis to identify genes associated with the brown midrib phenotype in the *bm6*_NIL. (a) Compared with B73, GO enrichment of up‐regulated genes in *bm6*_NIL and GO enrichment of down‐regulated genes in *bm6*_NIL. (b) Volcano map of differentially expressed genes of B73 and *bm6*_NIL. The horizontal coordinate indicates the logarithmic value of the multiple of the difference in gene expression levels between the two samples. The ordinate represents the negative pair value of FDR. (c) Heat maps of transcriptional levels of genes involved in lignin and flavone synthesis pathways in the B73 and *bm6*_NIL, gene names on the right. Red colour indicates the higher expression level. By contrast, the green indicates a lower expression level. (d) Compared with B73, KEGG enrichment of up‐regulated genes in *bm6*_NIL. (e) Compared with B73, KEGG enrichment of down‐regulated genes in *bm6*_ NIL. (f) qRT‐PCR was employed to validate the relative expression levels of selected genes identified in the transcriptome analysis. The mRNA level was normalized to that of *Actin*. Error bars represent the ± standard deviation of three biological replicates (**P < 0.01, ***P < 0.001).

We identified differentially expressed genes (DEGs) by comparing gene expression between B73 and *bm6*_NIL (|log_2_FoldChange| ≥ 1). A total of 3092 DEGs were found, with 2618 upregulated and 474 downregulated in *bm6*_NIL compared to B73 (Figure 2b, Table S1). Gene ontology (GO) analysis of DEGs revealed that down‐regulated genes in *bm6*_NIL were mainly involved in processes such as regulation of transcription by RNA polymerase II, DNA‐templated transcription, RNA biosynthesis, macromolecule biosynthesis, cellular biosynthesis, RNA metabolism, nucleobase‐containing compound metabolism and cellular metabolism, indicating a general decrease in biosynthetic activity. Conversely, up‐regulated genes in *bm6*_NIL were enriched in pathways related to cell wall organization, polysaccharide metabolism, phenylpropanoid biosynthesis and metabolic process and xylan metabolism (Figure 2a, Table S2). Phenylpropanoid is a bioactive secondary metabolites synthesized by the essential amino acid phenylalanine in plants under the catalysis of phenylalanine ammonolysis enzyme PAL, including anthocyanins, proanthocyanidins, flavonoids, lignin, etc. (Fraser and Chapple, 2011).

Then we compared the differential expression of key genes involved in lignin and flavonoid biosynthesis pathways between B73 and *bm6*_NIL. The results were visualized using heat maps to highlight expression patterns (Figure 2c). Notably, the key genes in both the lignin and flavonoid biosynthesis pathways exhibited an overall up‐regulation. To validate the transcriptome results further, we performed qRT‐PCR analysis, which confirmed that the gene expression trends were consistent with those observed in the RNA‐Seq data (Figure 2f, Table S1).

KEGG analysis of downregulated genes in *bm6*_NIL leaves showed that the only significantly enriched pathway is related to ABC transporters (Figure 2e, Table S3), which is crucial for transporting various molecules across cellular membranes, including the transport of secondary metabolites like lignin precursors (Alejandro *et al*., 2012; Panikashvili *et al*., 2010; Shiono *et al*., 2014). KEGG analysis of upregulated genes in *bm6*_NIL showed enrichment in pathways such as phenylpropanoid biosynthesis, plant secondary metabolites, motor proteins, photosynthesis‐antenna proteins, phenylalanine metabolism, benzoxazinoid biosynthesis and brassinosteroid biosynthesis (Figure 2d, Table S3).

### Untargeted metabolomics analysis

**Figure 3:**
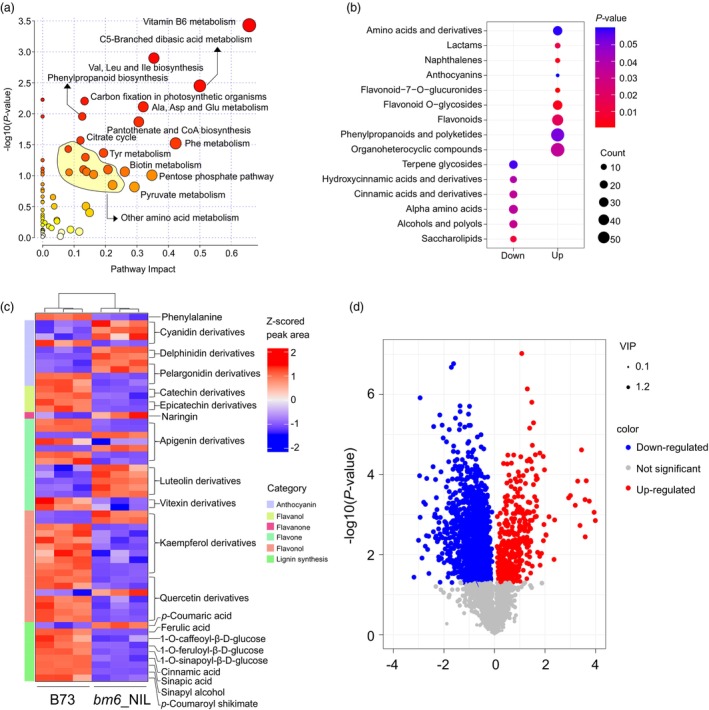
The *bm6_*NIL mutation redirects phenylpropanoid metabolic flux towards anthocyanin synthesis. (a) KEGG pathway analysis of differentially abundant metabolites (DAMs) between *bm6* and B73. The *x*‐axis represents pathway impact, which indicates the significance of each pathway based on the metabolites involved. The *y*‐axis shows the −log_10_ (*P*‐value), indicating the statistical significance of pathway enrichment. The size of each dot reflects the pathway impact, while the colour intensity (from yellow to red) represents the significance level, with red indicates higher significance. (b) Classyfire classification enrichment analysis of DAMs between *bm6* and B73, showing both up‐regulated and down‐regulated metabolites. The *x*‐axis categorizes metabolites as either ‘Up’ (up‐regulated) or ‘Down’ (down‐regulated), whereas the *y*‐axis shows the different compound classes. Dot colour indicates the *P*‐value, with purple represents higher significance, and the dot size represents the count of metabolites in each class. (c) Heatmap of phenylpropanoid metabolites in DAMs. The colour scale represents *Z*‐scored peak area, with red indicating higher intensity and blue indicating lower intensity. Metabolites are grouped based on metaboloite backbone category or function, such as anthocyanins, flavanols and lignin synthesis‐related compounds, which are indicated by the colour‐coded labels on the right side of the heatmap. (d) Volcano plot of differential metabolites. The *x*‐axis represents the log_2_ (fold change) of DAMs, with positive values indicating upregulation and negative values indicating down‐regulation. The *y*‐axis displays the −log_10_ (*P*‐value), representing the statistical significance of each metabolite. Metabolites with significant up‐regulation are marked in red, while significantly down‐regulated metabolites are shown in blue. Grey points indicate metabolites that are not significantly different. Point size reflects the VIP (Variable Importance in Projection) score, with larger points indicating higher VIP values, as indicated in the legend on the right.

We identified a total of 3346 non‐redundant features, among which 182 metabolites were precisely annotated based on MS^2^ spectra, whereas 3152 were annotated using only MS^1^ information (Table S7). Metabolite classification based on ClassyFire database (Feunang *et al*., 2016) revealed that the majority of the annotated metabolites fell under the superclasses of lipids and lipid‐like molecules, organoheterocyclic compounds, phenylpropanoids and polyketides and organic acids and derivatives (Figure S7a, Table S5). In terms of class‐level categorization, most metabolites belonged to carboxylic acids and derivatives, organooxygen compounds, flavonoids, benzene and substituted derivatives, prenol lipids and fatty acyls (Figure S7b, Table S5). We conducted differential metabolite analysis between the *bm6* mutant and B73 using a two‐tailed Student's *t*‐test, which resulted in the identification of 1597 differentially abundant metabolites (DAMs). Among these, 318 were up‐regulated, and 1279 were down‐regulated (Figure 3d). KEGG pathway analysis indicated that these DAMs primarily affected amino acid metabolism and carbohydrate metabolism pathways. Additionally, phenylpropanoid metabolism driven by phenylalanine was significantly impacted (Figure 3a,b).

To further explore the metabolic impact of the *bm6* mutation, we performed metabolite enrichment analysis using MetMiner (Wang *et al*., 2024a) on both up‐regulated and down‐regulated DAMs. The results showed that the down‐regulated metabolites were significantly enriched in categories such as cinnamic acids and derivatives, hydroxycinnamic acids and derivatives, terpene glycosides, alpha‐amino acids, alcohols and polyols and saccharolipids. In contrast, the up‐regulated metabolites were enriched in amino acids and derivatives, lactams, naphthalenes, anthocyanins, flavonoid‐7‐O‐glucuronides, flavonoid O‐glycosides, flavonoids, phenylpropanoids and polyketides and organoheterocyclic compounds. These findings suggest that, following the *bm6* mutation, phenylpropanoids, particularly flavonoids and anthocyanins, were significantly upregulated, while cinnamic acid derivatives were notably downregulated (Figure 3b). To confirm these results, we further examined the expression profiles of phenylpropanoids within the DAMs. We screened out typical phenylpropanoid metabolites, including those contributing to the lignin synthesis pathway, such as *p*‐coumaric acid, ferulic acid, cinnamic acid, sinapic acid and their derivatives, as well as those involved in the flavonoid biosynthesis pathway, including flavanones (naringin), flavones (apigenin and luteolin derivatives), flavonols (kaempferol and quercetin derivatives), flavanols (catechin and epicatechin derivatives) and anthocyanin derivatives such as cyanidin, delphinidin and pelargonidin (Figure 3c). Except for *p*‐coumaric acid, which was up‐regulated, other metabolites related to lignin synthesis were significantly down‐regulated. Meanwhile, flavonoid content increased significantly (Figure S6c), most anthocyanins were markedly upregulated. These results suggest that the *bm6* mutation inhibits the lignin biosynthesis pathway while simultaneously promoting the flavonoid biosynthesis pathway.

### ZmGCH1 restricts the activity of chalcone synthase by interacting with ZmPEBP15

**Figure 4:**
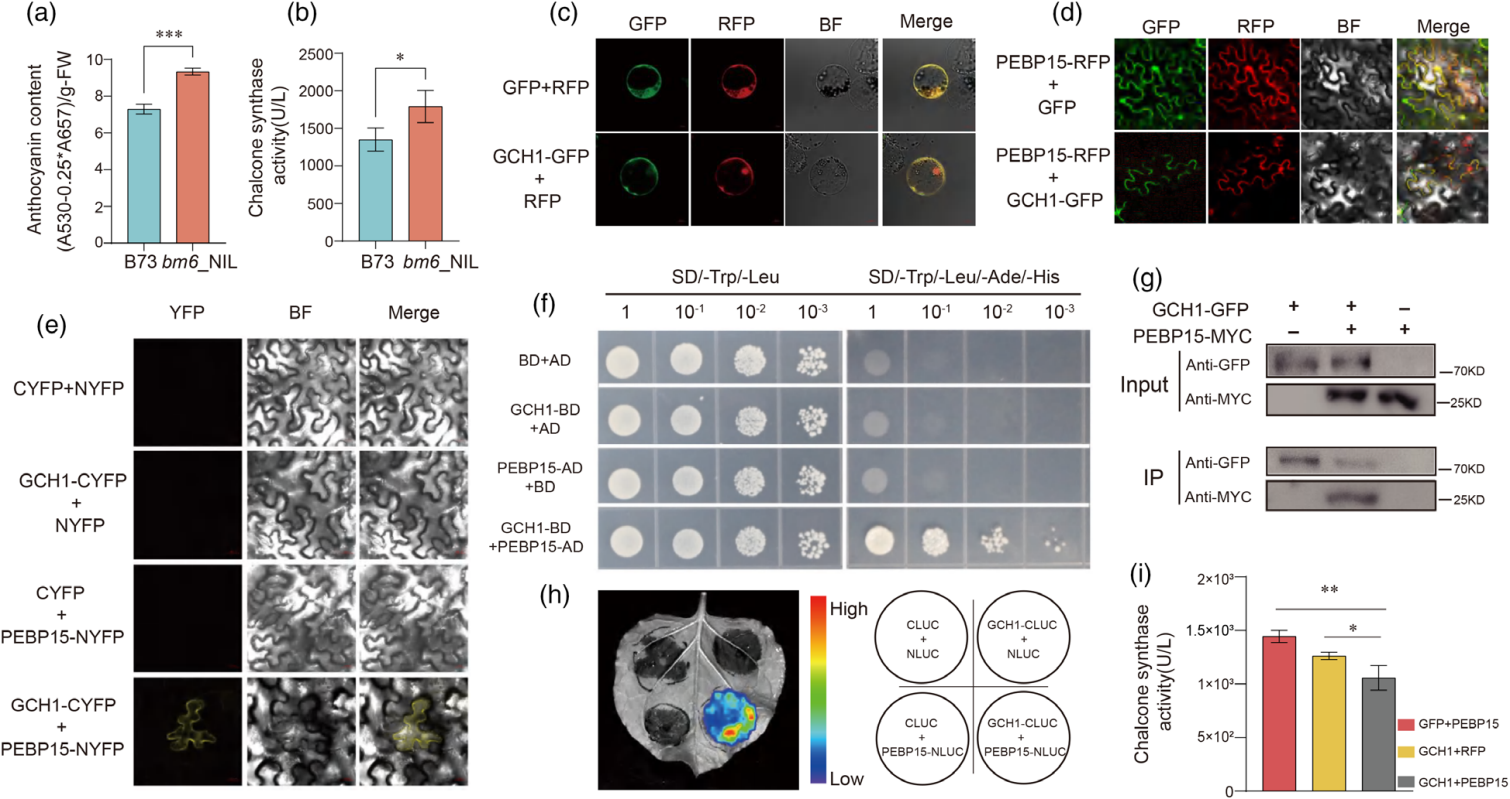
Interaction between ZmGCH1 protein and ZmPEBP15 protein. (a) Relative anthocyanin contents in leaves of B73 and *bm6*_NIL. (b) The chalcone synthase activity of leaves in B73 and *bm6*_NIL. (c) Subcellular localization of ZmGCH1 protein in maize protoplasts. Bars = 5 μm. (d) The fusion protein of ZmGCH1 and GFP and the fusion protein of PEBP15 and mCherry were co‐located in tobacco leaves. Bars = 20 μm. (e) BIFC assay shows the interaction between ZmGCH1 and ZmPEBP15. Bars = 50 μm. (f) Y2H assay shows the interaction between ZmGCH1 and ZmPEBP15. Serial dilutions (1:1, 1:10, 1:100, 1:1000) of transformed yeast cells were grown on the SD/−Leu/−Trp and SD/− Leu/−Trp/–His/−Ade medium. (g) Co‐IP assay of the interaction of ZmGCH1 and ZmPEBP15 in plant. Proteins were extracted from the protoplast of Maize co‐infiltrated with constructs expressing ZmGCH1‐GFP and ZmPEBP15‐MYC. (h) LIC assay showing the interaction between ZmGCH1 and ZmPEBP15. The LUC intensity was measured through co‐expression of different combinations. Blue, weak LUC intensity; Red, strong LUC intensity. (i) The chalcone synthase activity of different combinations was determined by transient transformation of tobacco. Error bars represent the ± standard deviation of three biological replicates (0.01 < *P < 0.05, **P < 0.01, ***P < 0.001).

We found that chalcone synthase activity was significantly increased in *bm6*_NIL (Figure 4b), which may explain why the common substrate of lignin and flavonoids is preferentially diverted to anthocyanin synthesis in *bm6*_NIL (Hoffmann *et al*., 2004). To elucidate how Zm*GCH1* mutations enhance the diversion of *p*‐coumaric acid into flavonoid biosynthesis pathways, we performed immunoprecipitation‐tandem mass spectrometry (IP‐MS) to identify the interaction protein of ZmGCH1. Using an anti‐GFP antibody, we purified ZmGCH1‐GFP and its interacting proteins in maize protoplasts. Among the potential interactors of ZmGCH1, we identified ZmPEBP15, a phosphatidylethanolamine‐binding protein (Figure S6d, Table S8). The expression of *ZmPEBP15* was significantly reduced in *bm6*_NIL (Figure S8a,b, Table S1).

We confirmed the co‐localization of ZmGCH1‐GFP and ZmPEBP15‐mCherry in the cytoplasm through transient assay in tobacco leaves (Figure 4d). The interaction between ZmGCH1 and ZmPEBP15 was further verified both in vitro and in vivo using bimolecular fluorescence complementation (Figure 4e), yeast two‐hybrid assays (Figure 4f), co‐immunoprecipitation (Figure 4g) and luciferase complementation assays (Figure 4h).

In addition, we utilized AlphaFold3 to predict the structural interactions between ZmGCH1 and ZmPEBP15. Our analysis revealed the presence of five strong hydrogen bonds formed between the two proteins, which play a crucial role in stabilizing their interaction. However, upon mutation of ZmGCH1, these hydrogen bonds were disrupted, leading to a loss of the interaction between the two proteins. This finding suggests that the integrity of these hydrogen bonds is essential for the functional interaction between ZmGCH1 and ZmPEBP15 and this disruption may impact chalcone synthase activity (Figure S9). We investigated the impact of various protein combinations on chalcone synthase activity through transient assay in tobacco. The results showed that the interaction between ZmGCH1 and ZmPEBP15 could partially reduce CHS enzyme activity (Figure 4i).

These findings suggest that the interaction between ZmGCH1 and PEBP15 may restrict CHS enzyme activity, thereby maintaining the balanced distribution of *p*‐coumaric acid. Upon disruption of this interaction due to the ZmGCH1 mutation, CHS activity increases, leading to a greater allocation of *p*‐coumaric acid towards the flavonoid biosynthesis pathway, resulting in the enhanced production of flavonoids (Figure S6c), especially the content of anthocyanins (Figure 4a).

### The introduction of *bm6* mutants leads to alterations in agronomic traits in Zhengdan 958 and Xianyu 335

To comprehensively evaluate the impact of the *bm6* mutation on lignin monomer content and agronomic traits, we introduced this mutation into two maize varieties, Zhengdan 958 and Xianyu 335 and assessed its potential for silage maize improvement (Figure S10). Field trials were meticulously designed and conducted across two geographically and climatically distinct locations to account for environmental variability: Tai'an (116°20′–117°59′E, 35°38′–36°28′N) and Suihua (124°13′–128°30′E, 45°03′–48°02′N).

**Figure 5:**
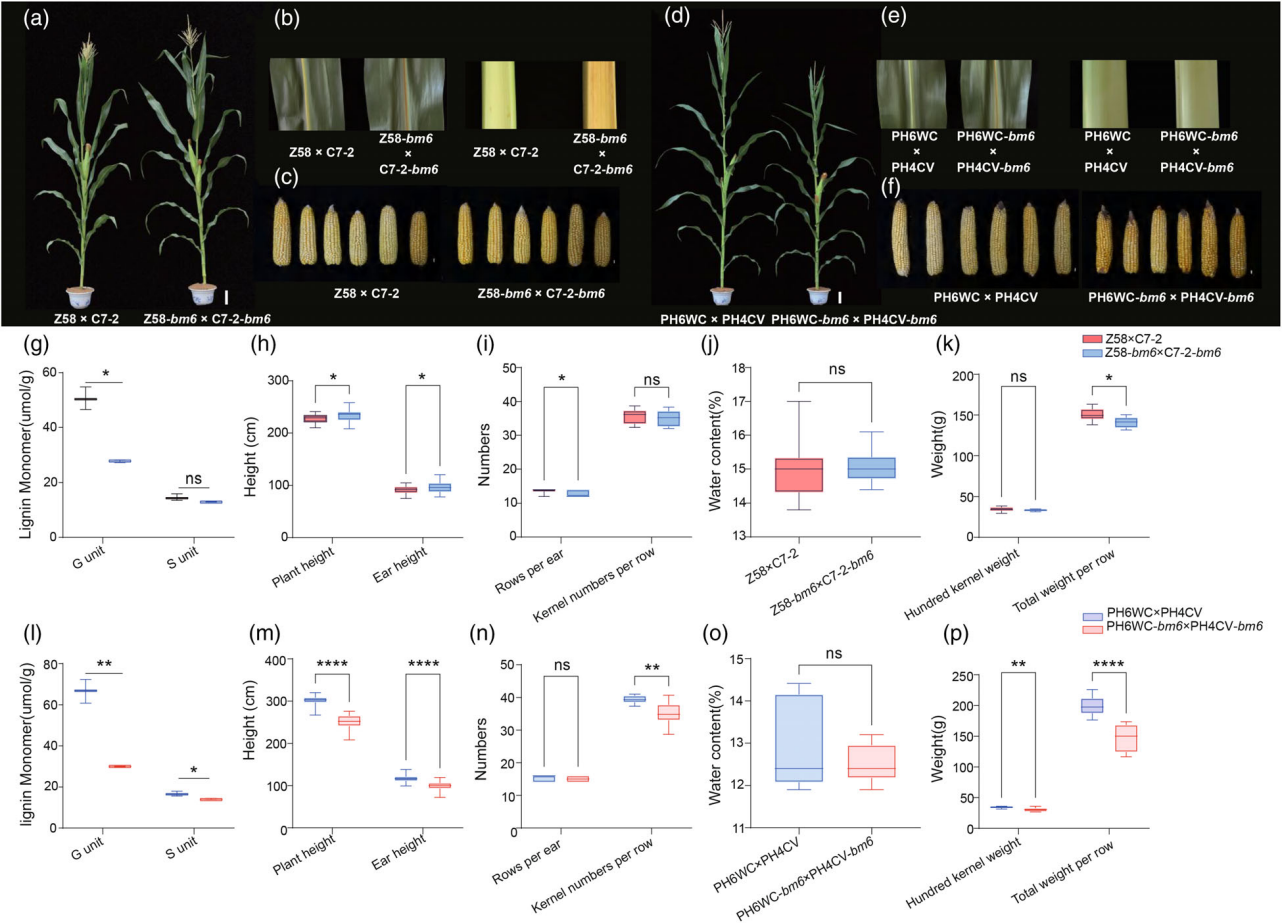
The introduction of the *bm6* gene resulted in changes to the agronomic characteristics of Zhengdan 958 and Xianyu 335. Error bars represent the ± standard deviation of three biological replicates (nsP >0.05, 0.01 < *P < 0.05, **P < 0.01, ***P < 0.001, ****P < 0.0001). (a) Comparison of whole corn between Zhengdan 958 and Zhengdan 958 after *bm6* gene introduction. Bar = 15 cm. (b) Comparison midrib and stem between Zhengdan 958 and Zhengdan 958 after *bm6* gene introduction. The left is the midrib of the leaf and the right is the stem tissue. (c) Comparison ear 40 days after pollination between Zhengdan 958 and Zhengdan 958 after *bm6* gene introduction. Bar = 1 cm. (d) Comparison whole corn between Xianyu 335 and Xianyu 335 after *bm6* gene introduction. Bar = 15 cm. (e) Comparison midrib and stem between Xianyu 335 and Xianyu 335 after *bm6* gene introduction. The left is the midrib of the leaf and the right is the stem tissue. (f) Comparison ear 40 days after pollination between Xianyu 335 and the Xianyu 335 after *bm6* gene introduction. Bar = 1 cm. (g) The contents of lignin monomer in the leaves of Zhengdan 958 after *bm6* gene and normal Zhengdan 958 were determined, respectively. (h) Comparison of plant height and ear height between Zhengdan 958 and Zhengdan 958 with *bm6* gene. (i) Comparison of rows per ear and kernel numbers per row between Zhengdan 958 and Zhengdan 958 after *bm6* gene introduction. (j) Comparison of grain water content between normal Zhengdan 958 and Zhengdan 958 after *bm6* gene introduction. (k) Comparison of hundred kernel weight and total weight per ear between Zhengdan 958 and Zhengdan 958 after *bm6* gene introduction. (l) The contents of lignin monomer in the leaves of Xianyu 335 after *bm6* gene and normal Xianyu 335 were determined, respectively. (m) Comparison of plant height and ear height between Xianyu 335 and Xianyu 335 with *bm6* gene. (n) Comparison of rows per ear and kernel numbers per row between Xianyu 335 and Xianyu 335 after *bm6* gene introduction. (o) Comparison of grain water content between normal Xianyu 335 and Xianyu 335 after *bm6* gene introduction. (p) Comparison of hundred kernel weight and total weight per ear between Xianyu 335 and Xianyu 335 after *bm6* gene introduction.

In Tai'an, agronomic traits were systematically analysed during the silking stage, a critical period for determining reproductive success and yield potential. In Zhengdan 958, the *bm6* mutation caused a pronounced brown midvein phenotype in leaves, a hallmark feature observed in *bm6*_NIL plants (Figure 5a,b). This visible browning is indicative of alterations in lignin deposition, specifically a significant reduction in the G lignin monomer content (Figure 5g). In addition to the leaf phenotype, the stems of Zhengdan 958 also exhibited a brown coloration (Figure 5b), further signifying reduced lignin accumulation in these tissues. Morphologically, the *bm6* mutation increased both plant and ear height but reduced the row number per ear, leading to a decrease in panicle weight (Figure 5c,h–k). In contrast, the response of Xianyu 335 to the *bm6* mutation displayed distinct differences, reflecting the influence of genetic background on phenotypic expression. Agronomically, Xianyu 335 experienced more pronounced adverse effects. Key traits, including plant height, ear height, kernel numbers per ear and hundred‐grain weight, were significantly reduced. The most consequential impact was observed in grain production, with an approximate 25% reduction in total grain weight per panicle (Figure 5f–p). Unlike Zhengdan 958, Xianyu 335 did not exhibit brown stems, indicating no significant reduction in lignin content within this tissue type. However, the leaves of Xianyu 335 displayed a phenotype similar to *bm6*_NIL, with reductions observed in both G and S lignin monomer contents (Figure 5d,e,l). These changes suggest that the mutation selectively affects lignin biosynthesis in certain tissues, depending on the variety.

In Suihua, the *bm6* mutation had minimal effects on Zhengdan 958, with no significant differences in agronomic traits compared to the conventional variety (Figure S11a–d). Traits such as plant height, ear height, row number per ear and grain weight remained comparable, suggesting that this variety exhibited a high degree of resilience to the mutation under the cooler and shorter growing season. For Xianyu 335, the only significant changes observed in agronomic traits were in plant height and ear height, indicating that the *bm6* mutation has a region‐specific effect on this variety (Figure S11e–h). The bm6 mutant exhibits a dwarf phenotype, whereas the introduction of Zhengdan 958 results in an increased plant height. To eliminate the possibility that this variation is due to heterozygous parental background, we conducted a parental recovery rate analysis. Our findings indicate that the recovery rates for Zheng 58 and Chang7‐2 were 92% and 91.4% (Figure S12a–f), respectively. This suggests that the observed increase in plant height following the introduction may be attributable to varietal characteristics or other factors, rather than heterozygosity in the parental background.

### *bm6* significantly increases the digestibility of Zhengdan 958 in the rumen of cattle in vitro

The compact structure and complex chemical composition of plant cell walls hinder enzymatic digestion, known as the biomass anti‐degradation barrier (Zhao *et al*., 2012). In ruminant feed, high lignin content reduces digestibility and increases energy consumption. Total lignin levels and the ratio of lignin monomers, particularly syringyl (S) to guaiacyl (G) units are key factors affecting cell wall degradability. The S/G ratio influences carbohydrate accessibility, impacting digestibility and nutrient availability in forage crops for animal feed (Argillier *et al*., 1996; Fontaine *et al*., 2003; Grabber *et al*., 2004).

**Figure 6:**
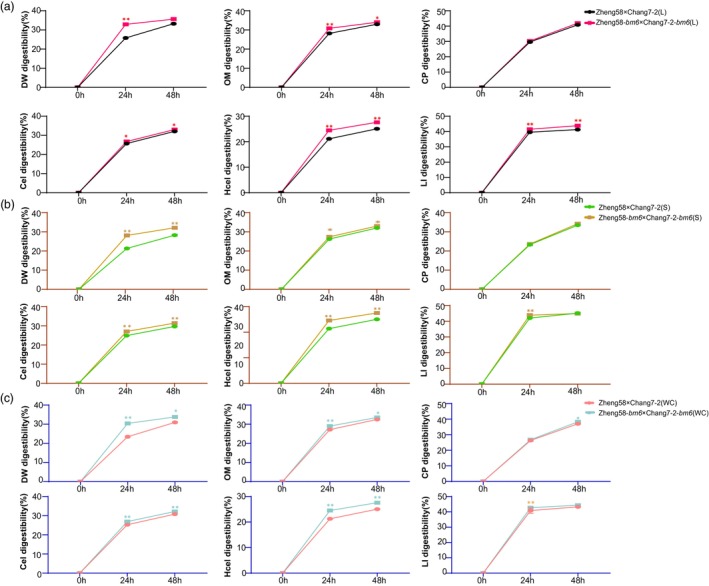
*bm6* significantly increased the degradation rate of Zhengdan 958 maize plants in the rumen of cattle in vitro. (a) Changes of 48 h digestibility of dry matter (DM), organic matter (OM), crude protein (CP), cellulose (Cel), hemicellulose (Hcel) and lignin (LI) in leaves of Zhengdan 958 after *bm6* gene introduction in bovine rumen. (b) Changes of 48 h digestibility of dry matter (DM), organic matter (OM), crude protein (CP), cellulose (Cel), hemicellulose (Hcel) and lignin (LI) in stem(S) of Zhengdan 958 after *bm6* gene introduction in bovine rumen. (c) Changes of 48 h digestibility of dry matter (DM), organic matter (OM), crude protein (CP), cellulose (Cel), hemicellulose (Hcel) and lignin (LI) in whole corn (WC) of Zhengdan 958 after *bm6* gene introduction in bovine rumen. Error bars represent the ± standard deviation of three biological replicates (nsP >0.05, 0.01 < *P < 0.05, **P < 0.01, ***P < 0.001, ****P < 0.0001).

Considering the impact on agronomic traits of *bm6* mutation, Zhengdan 958, which exhibited a relatively mild response to the mutation, was chosen for the degradation rates test using a rumen in vitro system. We conducted experiments on Zhengdan958 and Zhengdan958 plants introduced with *bm6*, respectively, to measure the degradation rates of various components in leaves, stems, and the whole plant in vitro (Figure 6). Compared to Zhengdan 958 leaves (L), the degradation rates of dry matter (DW), organic matter (OM), cellulose (Cel), hemicellulose (Hcel) and lignin (LI) significantly increased after 24 h treatment in Zhengdan 958 plants introduced with *bm6* (Figure 6a). Astonishingly, after 48 h of treatment, the degradation rates of OM, Cel, Hcel and LI, except dry weight, still exhibited significant increases (Figure 6a).

Similarly, compared to Zhengdan 958 stems (S), plants modified with *bm6* significantly enhanced the degradation rates of DW, OM, Cel, Hcel and LI at 24 h (Figure 6b), and after 48 h, the degradation rates of DW, OM, Cel and Hcel remained markedly elevated (Figure 6b). In comparison to Zhengdan 958 whole plants (WC), Zheng58‐*bm6*×Chang7‐2‐*bm6* plants exhibited significantly increased degradation rates of DW, OM, Cel, Hcel and LI at 24 h (Figure 6c), after 48 h treatment, the degradation rates of DW, OM, crude protein (CP), Cel and Hcel also demonstrated significant enhancements. These results demonstrate that the introduction of *bm6* significantly increases the degradation rates of leaves, stems, and the whole plant in Zhengdan 958, highlighting the potential of *bm6* to improve maize as a storage feed. This enhancement suggests promising applications for developing better feed options for livestock, offering a valuable approach to optimizing feed efficiency.

## Discussion

**Figure 7:**
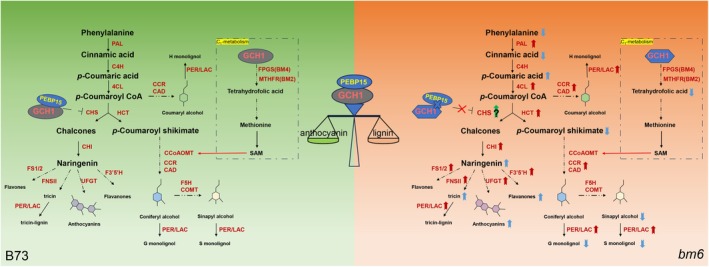
Differential changes of lignin and anthocyanin synthesis pathways in B73 and *bm6*. Black text denotes metabolites, red text denotes genes, and blue arrows indicate changes in metabolite content, with upward arrows representing increases and downward arrows representing decreases. Red arrows indicate gene transcription levels, with upward arrows for increases and downward arrows for decreases, whereas a green upward arrow indicates increased enzyme activity. PAL: phenylalanine ammonia lyase; C4H: cinnamate 4‐hydroxylase; 4CL: 4‐coumarate:CoA ligase; CHS: chalcone synthase; CHI: chalcone isomerase; FSI/FS2: flavone synthase; F3′5′H: flavonoid 3′5′hydroxylase; UFGT: UDP flavonoid glucosyl transferase; HCT: hydroxycinnamoyl transferase; CCR: (hydroxy) cinnamoyl CoA reductase; CAD: (hydroxy) cinnamyl alcohol dehydrogenase; CCoAOMT: caffeoyl CoA O‐methyltransferase; F5H: ferulate‐5‐hydroxylase; COMT: caffeic acid (5‐hydroxy‐coniferaldehyde) O‐methyltransferase; FPGS: folylpolyglutamate synthetase; MTHFR: methylenetetrahydrofolate reductase; PER: peroxidases; LAC: laccases.

*ZmGCH1* is a critical enzyme involved in the one‐carbon unit transfer reaction, an essential biochemical process in various metabolic pathways, including the synthesis of tetrahydrofolate (Cossins and Chen, 1997; Hanson *et al*., 2000). This gene was first identified as a novel maize *brown midrib* (*bm*) gene through the efforts of our research collaborator (Chen *et al*., 2012). After discovering the mutant phenotype, we carried out extensive fine mapping using a range of genetic markers to narrow down the region of interest. This work ultimately led to the identification of *ZmGCH1* as the gene responsible for the *bm6* phenotype. Our findings align closely with those reported by Leonard *et al*. (2022) further validating the location of the *BM6* locus. In the one‐carbon metabolic pathway, S‐adenosylmethionine (SAM) is produced through a multi‐stage enzyme‐linked reaction involving tetrahydrofolate. SAM provides methyl units as active methyl donors for the catalytic steps of COMT and CCoAOMT, which are required for the synthesis of G and S lignin. Metabolome data showed a significant reduction in tetrahydrofolate in *bm6*_NIL (Tables S4 and S6), indicating that the *bm6* gene mutation affects the one‐carbon metabolic pathway. However, transcriptome and metabolome analyses revealed no significant changes in the transcription levels of key enzymes such as FPGS and MTHFR, and no differences in key metabolites such as methionine and SAM in the one‐carbon metabolic pathway (Figure 7). This observation suggests that the reduction in tetrahydrofolate alone cannot fully explain the reduction in lignin monomers, and other factors may also contribute to this phenotype.

In the *bm6*_NIL mutant, phenylalanine and cinnamic acid levels significantly decreased, while *p*‐coumaric acid accumulated (Figure 3c). The elevated expression of PALs and 4CL may accelerate the conversion of phenylalanine to cinnamic acid and cinnamic acid to *p*‐coumaric acid, respectively (Figure 2c,f). As a precursor for lignin and anthocyanin biosynthesis, *p*‐coumaric acid is directed by HCT and CHS. *p*‐Coumaroyl acid is converted into *p*‐Coumaroyl shikimate under the action of HCT (Fraser and Chapple, 2011).

Metabolome analysis revealed significant increases in proanthocyanidins, naringin and anthocyanins, which provided indirect evidence for the increase of naringenin content (Figures 3c and 4a). While key lignin pathway enzymes (HCT, CCR, CAD) were up‐regulated, sinapyl alcohol, a lignin precursor, was reduced (Figures 2c,f and 3c). These results suggest that mutations in the *ZmGCH1* gene, which lead to a reduction in tetrahydrofolate, may alter the availability of methyl donors, thereby reducing lignin monomer production. Additionally, this shifts more shared precursors in the phenylpropanoid pathway towards flavonoid synthesis, increasing anthocyanin content and decreasing lignin monomer levels.

To further explore the underlying causes for the increased diversion of *p*‐coumaric acid into the flavonoid biosynthesis pathway, additional analyses were conducted. Through IP‐MS screening, we identified ZmPEBP15, which belongs to the phosphatidylethanolamine‐binding protein family, interacting with ZmGCH1. (Figure S6d, Table S8). We proved that there was a strong interaction between the two proteins by yeast two‐hybrid experiment, luciferase complementary experiment, BIFC experiment and Co‐IP experiment (Figure 4e–h). AlphaFold3 (Abramson *et al*., 2024) was also used to predict the protein structure of the two, and the interaction sites between the two were analysed. It was found that there were hydrogen bonds at five positions: 203Asn‐139Arg, 202Glu‐139Arg, 325Asn‐132Thr, 317Cys‐134Tyr, 394His‐151Tyr (Figure S9a,b). Interestingly, structural analysis of the mutated bm6 protein revealed the disappearance of these hydrogen bonds. This suggests that in the *bm6* mutant, the protein interaction between ZmGCH1 and ZmPEBP15 is disrupted (Figure S9c–g). The establishment and dissolution of these interactions may influence the distribution of precursor substances.

Chalcone synthase has been reported as the key enzyme that directs *p*‐coumaric acid into the flavonoid biosynthesis pathway (Sébastien *et al*., 2007). Higher CHS activity channels more *p*‐coumaric acid toward anthocyanin synthesis, whereas lower CHS activity may result in greater allocation of *p*‐coumaric acid to lignin biosynthesis (Fraser and Chapple, 2011; Sébastien *et al*., 2007; van den Hof *et al*., 2008). Chalcone synthase activity in *bm6*_NIL was significantly higher compared to B73 (Figure 4b). We assessed CHS activity in tobacco in the presence of ZmGCH1 and ZmPEBP15 individually or together. The results demonstrated that their interaction partially restricted CHS activity (Figure 4i), potentially helping to maintain the balance between lignin and flavonoid biosynthesis. When this interaction was disrupted, the regulatory constraint on CHS was lifted, allowing CHS to function at higher levels. This led to a significant increase in CHS activity, redirecting more metabolic intermediates toward the flavonoid biosynthesis pathway (Figure 4b). Lignin is primarily synthesized through the condensation of three monomeric precursors, known as monolignols, which are alcohols derived from phenylpropanoid metabolism. The monolignol is synthesized in the cytoplasm and subsequently transported to the cell wall, where it polymerizes via free radical reactions. The formation of free radicals is mainly catalysed by peroxidase and laccase (Miao and Liu, 2010; Perkins *et al*., 2019; Väisänen *et al*., 2020). We observed elevated transcription levels of numerous PEXs and LACs genes (Figure S8c, Table S1), which may serve to accelerate the polymerization of the increased tricin, compensating for the reduction in G and S lignin monomers and thereby maintaining the stability of total lignin levels. Previous studies have shown that the transport of lignin precursors across the plasma membrane into the vacuole is an ATP‐dependent primary transport process, mediated by ATP‐binding cassette (ABC) transporters in Arabidopsis and rice (Bekaert *et al*., 2008; Holton and Cornish, 1995; Miao and Liu, 2010; Vermerris and Boon, 2001). Transcriptomic analysis of KEGG down‐regulated gene enrichment in the ABC transporter pathway suggests a potential link to the decrease in monolignols and the disruption of their transport (Figure 2e, Table S3).

Changes in lignin and anthocyanin content are of significant importance for storage feed quality. To assess the potential applications of *ZmGCH1*, we introduced the *bm6* mutation into two excellent maize varieties: Zhengdan 958 and Xianyu 335. Through a comparison of the improved varieties with the original varieties, we found that the gene had different effects on different maize varieties. The improved Zhengdan 958, which was slightly affected, was selected as a feed for in vitro rumen experiments to detect the effect of the *ZmGCH1* gene on digestibility. We found that the dry matter and crude protein of the improved varieties were more easily digested and absorbed, not only in the stems and leaves but also throughout the whole plant. Additionally, the reduction in lignin content can reduce the extraction cost of bioethanol and enable more efficient bioethanol production (Wang *et al*., 2024b). These results indicate that *bm6* can be utilized not only for improving storage feed quality but also for enhancing bioethanol production.

## Conclusion

In summary, the *bm6* mutant phenotype is attributed to a mutation in the *ZmGCH1* gene. In B73, ZmGCH1 plays a crucial role in tetrahydrofolate synthesis for lignin biosynthesis and interacts with ZmPEBP15 to regulate CHS enzyme activity, balancing lignin and anthocyanin pathways. In *bm6*, two amino acid changes in ZmGCH1 alter its conformation, disrupting its interaction with ZmPEBP15. This disruption leads to deregulated CHS activity, redirecting more *p*‐coumaric acid to flavonoid synthesis, resulting in reduced lignin content and increased anthocyanin accumulation. Our findings provide valuable strategies to optimize maize production for both food and livestock feed applications.

## Methods

### Plant materials and growth conditions

The materials used in the experiment were B73, *bm6* mutants and *bm6*_NIL. The *bm6* mutant was obtained from maize genetic cooperative inventory centre (http://maizecoop.cropsci.uiuc.edu/). The *bm6*_NIL isogenic line was created in our laboratory. BC_8_F_2_ population, generated by backcrossing *bm6* with B73, was used for genetic mapping. *bm6* mutant was crossbred with B73 to obtain F1 generation, and B73 was used as recurrent parent backcross for eight generations to obtain BC_8_F_1_. The principle was the same as the *bm6*_NIL material creation process, and BC_8_F_2_ isolated population was obtained by self‐crossing on the basis of BC_8_F_1_. *bm6*_NIL of BC_3_F_2_ generation from Zheng 58, Chang 7–2, PH6WC and PH4CV background were obtained in the same way. Maize materials were grown in experimental fields in Tai'an, Harbin and Sanya.

### RNA‐sequencing and qRT‐PCR analysis

Raw data were filtered using fastp (v0.23.2) to remove low‐quality sequences, and clean reads were used for transcriptome analysis. STAR (v2.7.10b) aligned reads to the B73 reference genome (Zm‐B73‐REFERENCE‐NAM‐5.0). Gene expression quantification was performed using feature Counts (v2.0.6). Gene expression levels were normalized using the FPKM (Fragments Per Kilobase of transcript per Million mapped reads) method. DEGs across all groups were identified using the edgeR software package (http://www.r‐project.org/). DEGs were considered significant if |log_2_FoldChange| ≥ 1.

For RNA‐sequencing analysis, we randomly clipped the veins of B73 and *bm6*_NIL at V8 stage for liquid nitrogen grinding. Total RNA was extracted using the Ultrapure RNA kit (CWBIO), part of it was sent to the BGI company for sequencing, the other part was reverse‐transcribed into cDNA using HiScript II Q RT SuperMix for qPCR kit (Vazyme). qRT‐PCR was employed to quantify the expression of selected genes, utilizing the Hieff TM qPCR SYBR Green Master Mix (YEASEN). Experiments were biological replicates at least three times, with primers listed in Table S9.

### Degradation rates test of maize using a rumen in vitro system

Rumen fluid was collected from three Simmental cattle with permanent rumen fistulas, 1 h before morning feeding. It was filtered through four layers of gauze, stored in preheated anaerobic hot water bottles and quickly brought back to the laboratory. Preparation of artificial rumen fluid was done according to Menke *et al*. (1979). Samples, including whole plants, stems and leaves of both control and mutant groups (six replicates each), were pre‐weighed (1 g DM), put into polyester bags (size: 5 cm × 5 cm; diameter: 20 μm), sealed and placed in a 100 mL flask. Sixty millilitres of artificial rumen fluid preheated to 39 °C was added to the bottle and sealed with a plastic cap. The flask was then incubated in an incubator (TC‐111B; Tocan, Shanghai, China) at 125 rpm and samples were collected after 24 and 48 h.

The conventional nutrients, including dry matter, crude protein, crude ash and crude fibre, were determined before and after degradation. Dry matter was determined using the drying method (GB/T 6435‐2014), crude protein was measured by the semi‐micro Kjeldahl nitrogen determination method (GB/T 6432‐2018), and crude ash was quantified using the ashing method at 550°C (GB/T 6438‐2007). Normal form fibre determination method was used to determine the content of neutral detergent fibre (NDF), acid detergent fibre (ADF) and acid detergent lignin (ADL), according to the formula: Cellulose = ADF (%) − ADL (%), hemicellulose = DNF (%) − ADF (%), lignin = ADL (%) − ash to calculate the content of cellulose, hemicellulose and lignin.

### Determination of anthocyanins, lignin content and flavonoid content

Total anthocyanins were extracted by methanol–hydrochloric acid method (Mano *et al*., 2007; Rabino and Mancinelli, 1986). Approximately 0.2 g of sample was taken and incubated in 1 mL of acidic solution containing 80% methanol, 0.16% ascorbic acid, 0.16% butylhydroquinone and 0.1% hydrochloric acid in the dark for 18 h. After gentle shaking, the solution was centrifuged at 120 00 ***g*** for 2 min. Transfer the supernatant and measure the absorbance at 530 nm and 657 nm using ultraviolet spectrophotometry. The relative content of anthocyanins was calculated using the formula: (A530–0.25 × A657) × M^−1^. A530 and A657 refer to the absorbance values at the specified wavelengths, while M represents the fresh weight (in grams) of the plant tissue used for extraction. The total lignin content was determined by spectrophotometry. For specific steps, refer to the instructions of the detection kit for lignin content (Solarbio, Beijing, China). Flavonoid content was determined by enzyme‐labelled method. For specific steps, refer to the instructions of plant flavonoid kit (MICHY BIOLOGY).

### Untargeted metabolomics profiling using LC‐ESI‐HRMS

Metabolites extracted from maize leaf tissue were analysed using high‐resolution mass spectrometry with a Thermo Fisher Vanquish Flex UHPLC system coupled to a Quadrupole‐Orbitrap Exploris 240 mass spectrometer (Thermo Fisher Scientific, Waltham, MA). The Thermo Fisher LC‐ESI‐HRMS was operated in full‐scan, data‐dependent MS/MS acquisition mode in both positive and negative modes. The Instrumental conditions were set as follows. UHPLC: the column used was a Thermo Scientific™ Hypersil Gold Vanquish (100 × 2.1 mm, 1.9 μm); solvent system: 0.1% formic acid in water (solution A) and acetonitrile (solution B); column temperature: 30°C. Mobile phase A consisted of HPLC‐grade H_2_O with 0.1% (v/v) formic acid (Merck, Germany), and mobile phase B consisted of HPLC‐grade acetonitrile (Merck, Germany). The gradient elution conditions were as follows: from 0 to 2 min, mobile phase B was maintained at 10%; from 2 to 10 min, it increased to 50%; from 10.1 to 13 min, mobile phase B was held at 80%; from 13 to 14 min, it increased to 95%; from 14 to 14.1 min, mobile phase B decreased to 10% and from 14.1 to 18 min, it was maintained at 10%. The flow rate was set to 0.3 mL/min. MS detection parameters were as follows: spray voltage, +3.5 kV/−3.2 kV; capillary temperature: 320°C; sheath gas: 35 arb; AUX gas: 15 arb; AUX gas heater temperature: 350°C; s‐lens RF level: 50%; scan range: *m*/*z* 70–1050; resolution: 70 000 (MS1) and 17 500 (MS2); stepped normalized collision energy (NCE): 20, 40 and 60.

### Yeast two‐hybrid assay

The yeast two‐hybrid experiments were conducted according to the Matchmaker Gold yeast two‐hybrid system, as detailed in the user manual (Clontech, Irvine, CA, USA). The CDS full‐length sequence of *ZmGCH1* was reassembled into *pGBKT7*, and the CDS full‐length sequence of *ZmPEBP15* was reassembled into *pGADT7*. The co‐transferred Y2H yeast strains were cultured on −Trp/−Leu medium and screened on −Trp/−Leu/‐His/−Ade medium.

### Luciferase complementation assay

The coding sequences of *ZmGCH1* and *ZmPEBP15* were separately cloned into luciferase reporter vectors to investigate their interaction through a luciferase complementation assay in *Nicotiana benthamiana* leaves. Specifically, the *ZmGCH1* coding sequence was inserted at the *Kpn*I and *Sal*I sites of the *JW772‐cLUC* vector, while *ZmPEBP15* was cloned into the *JW771‐nLUC* vector using the same restriction sites. Both constructs were transformed into *Agrobacterium tumefaciens* strain GV3101 via the freeze–thaw method for subsequent transient expression. After 48 h, the leaves were infiltrated with D‐Luciferin‐K + Salt Bioluminescent Substrate (XenoLight) to stimulate bioluminescence and luciferase (LUC) signals were imaged using a vivo imager.

### Subcellular localization analysis

Transient transformation was performed to investigate the subcellular localization of ZmGCH1 and ZmPEBP15 in tobacco leaves and maize protoplasts. The coding sequence of *ZmGCH1* was inserted between the *BamH*I and *Sal*I sites of the *pCAMBIA1300‐GFP* vector to construct the *ZmGCH1‐GFP* plasmid. The empty *pCAMBIA1300‐GFP* vector, along with the *ZmGCH1‐GFP* plasmid, was transformed into *Agrobacterium strain* GV3101 and used for transient transformation in *Nicotiana benthamiana*. After 48 h of culture, GFP fluorescence localization was observed using a confocal microscope (LSCM880; Carl Zeiss AG, Oberkochen, Germany). Similarly, the CDS of *ZmPEBP15* was inserted into the 35S promoter of the *pCAMBIA1300‐RFP* vector, creating the *ZmPEBP15‐RFP* plasmid, which was also transformed into GV3101. Co‐transformation of GV3101 with *ZmGCH1‐GFP* and ZmPEBP15‐RFP, and GV3101 with the empty *pCAMBIA1300‐GFP* vector and *ZmPEBP15‐RFP* was performed in *Nicotiana benthamiana*.

Maize protoplasts were isolated from B73 etiolated seedlings that had been cultured in the dark for 2 weeks. Approximately 2 μg/μl of the *ZmGCH1‐GFP* plasmid and 2 μg/μL of *pCAMBIA1300‐RFP* plasmid were transformed into 200 μL of protoplasts. After a 12 h dark incubation at room temperature, fluorescence signals were observed using a confocal microscope (LSCM880, Carl Zeiss AG, Germany).

### Immunoprecipitation‐tandem mass spectrometry analysis

The *ZmGCH1‐GFP* plasmid was transiently expressed in maize protoplasts, followed by extraction of total protein. Co‐immunoprecipitation (Co‐IP) was performed using an anti‐GFP antibody to isolate ZmGCH1‐GFP and its interacting proteins. The immunoprecipitated proteins were then subjected to IP‐MS for analysis, which was conducted by APTBIO, Shanghai, China. A total of 2715 proteins were identified in the IP group, with the majority (85.89%) exhibiting a UniquePepCount ranging from 1 to 5, while a smaller fraction (14.11%) showed a UniquePepCount exceeding 5. Given that the ZmGCH1 protein is localized in the cytoplasm, we initially excluded proteins associated with chloroplasts, mitochondria and nuclei from our analysis. To refine our selection, we applied a screening criterion of UniquePepCount >5, which identified several proteins of interest, including Chalcone‐flavanone isomerase and others. Using a web‐based tool, we generated protein interaction predictions and subsequently validated these interactions experimentally. Through this approach, we discovered that ZmPEBP15 interacts with our target protein, ZmGCH1.

### Bimolecular fluorescence complementation assay

BiFC analysis was conducted following the method described by Walter *et al*. (2004). The full‐length coding sequence (CDS) of *ZmGCH1* was subcloned into the *PUC‐SPYCE* vector, whereas the *ZmPEBP15* sequence was cloned into the *PUC‐SPYNE* vector. The resulting constructs were introduced into *Agrobacterium tumefaciens* strain GV3101, then co‐infiltrated into *Nicotiana benthamiana* leaves to assess potential protein–protein interactions. After infiltration, YFP fluorescence, indicative of BiFC interaction, was observed using a confocal laser scanning microscope (LSCM880; Carl Zeiss AG).

### Co‐immunoprecipitation and Western blotting analysis

The plasmids *ZmGCH1‐GFP* and *PEBP15‐MYC* were co‐transformed into maize protoplasts. After 48 h, total protein was extracted and incubated with GFP antibody‐conjugated beads for 2 h at 4 °C. The beads were washed five times to remove non‐specifically bound proteins. Bound proteins were then eluted, mixed with loading buffer, and boiled for 10 min. Western blotting was performed using anti‐GFP and anti‐MYC antibodies to detect ZmGCH1‐GFP and PEBP15‐MYC, respectively, followed by chemiluminescent.

### Enzyme activity assay

The enzyme activity of ZmGCH1 was measured using a double‐antibody sandwich enzyme‐linked immunosorbent assay (ELISA). Standard and sample solutions were added to an enzyme‐labelled plate, followed by the addition of a horseradish peroxidase‐conjugated detection antibody. The plate was incubated at 37°C for 60 min, followed by five washes with wash buffer. After adding the substrate, the plate was incubated at 37°C in the dark for 15 min. The optical density (OD) was measured at 450 nm. A standard curve was constructed based on the standard concentrations and corresponding OD values to determine the concentration of each sample. Refer to the plant ZmGCH1 ELISA Kit for detailed procedural steps (KEVINO).

The chalcone synthase activity assay was conducted following the instructions in the Plant Chalcone Synthase ELISA Kit (MEIMIAN). To examine the effects of ZmGCH1 and ZmPEBP15 on chalcone synthase activity, we assessed enzyme activity in vitro ZmGCH1‐GFP, the empty GFP vector, PEBP15‐MYC, and the empty MYC vector were individually transformed into *Agrobacterium tumefaciens* strain GV3101. After 12 h of culture and screening, the cells were centrifuged at 5000 rpm for 10 min, and the bacterial suspensions were adjusted to an OD_600_ = 1 with MMS solution. We then prepared three mixtures: ZmGCH1‐GFP with empty MYC, ZmGCH1‐GFP with PEBP15‐MYC and empty GFP with PEBP15‐MYC in equal volumes. These combinations were co‐injected into the same tobacco leaf. Leaf discs of equal size were harvested with a hole punch, ground and used to measure chalcone synthase activity.

## Author contributions

H.J.L, C.Z., X.R.Y. and X.S.Z. designed the study and conceived the project. T.L., X.B.Z., H.J.L. and C.C.X. provided constructive suggestions. M.Y.Z., X.H.L., Z.X.Z., J.X.Z., M.Y.L., Y.X.L., E.L.Q., Z.A.T. and M.F.S. performed the experiments. X.W., S.Z.J., J.L.Z. and H.J.Z. analysed data. C.Z. and M.Y.Z drafted the manuscript. H.J.L, C.Z., C.C.X., T.L. and X.Y.Z. revised the manuscript. All authors approved the manuscript.

## Conflict of interest

The authors declare no conflict of interest.

## Supporting information


**Figure S1.** Analysis of B73 and bm6_NIL phenotypic.

**Figure S2.** Molecular markers screening.

**Figure S3.** The expression profiles of the six candidate genes.

**Figure S4.** Quantitative analysis of fine location candidate genes and histological sections of B73 and bm6_NIL.

**Figure S5.** Tissue‐specific expression and phylogenetic tree analysis.

**Figure S6.** Subcellular localization and IP‐MS analysis.

**Figure S7.** Metabolite classification based on ClassyFire database.

**Figure S8.** Transcriptome and qRT‐PCR analysis.

**Figure S9.** Predicted interaction between ZmGCH1 protein and ZmPEBP15 protein using AlphaFold Server.

**Figure S10.** Field phenotypes.

**Figure S11.** The introduction of the bm6 gene resulted in changes to the agronomic traits of Zhengdan 958 and Xianyu 335 in Suihua city.

**Figure S12.** Analysis of the parental background recovery rate in Zhengdan 958 following the introduction of the bm6 Gene.

**Table S1.** Differentially expressed gene.
**Table S2.** GO enrichment result.
**Table S3.** KEGG enrichment result.
**Table S4.** Compound annotation.
**Table S5.** Compound classification.
**Table S6.** Different accumulated metabolites (DAMs) between bm6 and B73.
**Table S7.** Accumulation profile of non‐redundant compounds.
**Table S8.** IP‐MS result.
**Table S9.** Primer Sequences Used in the Study.

## Data Availability

Data supporting the finding of this work are available within the paper and its supplementary information files. The raw data generated from this study have been uploaded to the NCBI SRA (Sequence Read Archive) database and can be downloaded using the accession number: PRJNA1185680.
