# Supplementary figures and images for: Modulation of lignin and anthocyanin homeostasis by GTP cyclohydrolase1 in maize
Source: Plant Biotechnol J. 2025 Mar 28;23(7):2449–63. doi: 10.1111/pbi.70061 (PMC12205856; doi:10.1111/pbi.70061)

(a)

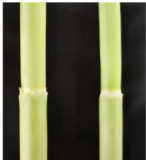

B73

*bm6\_NIL*

(b)

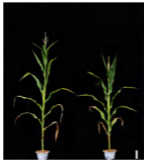

B73

*bm6\_NIL*

(c)

Stem

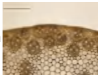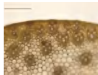

Root

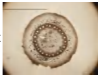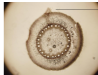

B73

*bm6\_NIL*

Supplement: Supplementary file 1 — Figure S1. Analysis of B73 and bm6_NIL phenotypic. [file PBI-23-2449-s005.pdf]

(a)

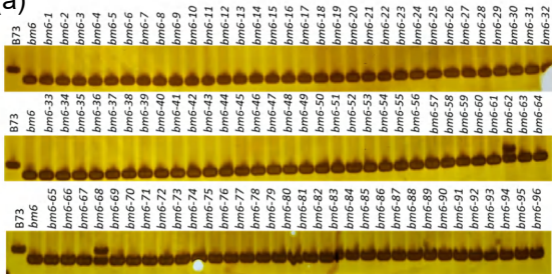

(b)

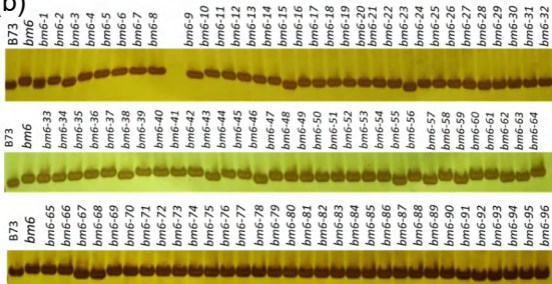

(c)

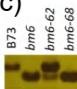

(d)

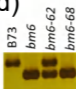

(e)

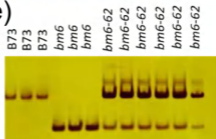

Supplement: Supplementary file 2 — Figure S2. Molecular markers screening. [file PBI-23-2449-s006.pdf]

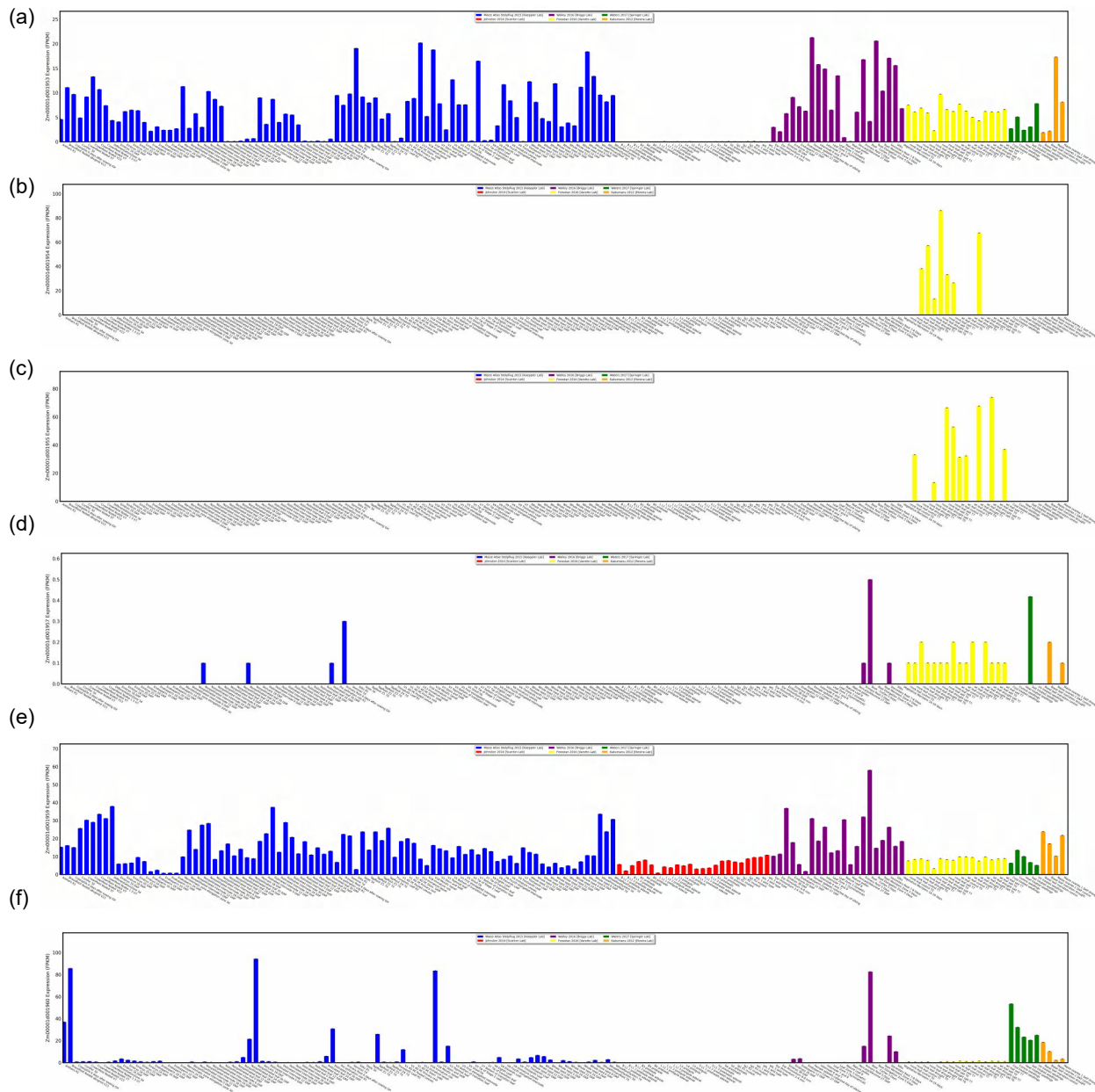

Supplement: Supplementary file 3 — Figure S3. The expression profiles of the six candidate genes. [file PBI-23-2449-s001.pdf]

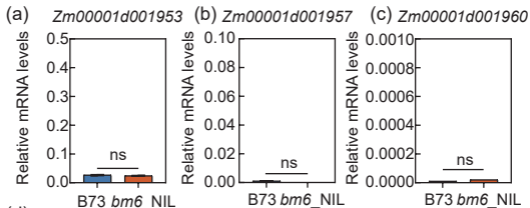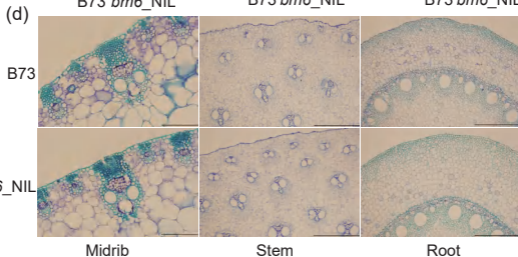

Supplement: Supplementary file 4 — Figure S4. Quantitative analysis of fine location candidate genes and histological sections of B73 and bm6_NIL. [file PBI-23-2449-s009.pdf]

(a)

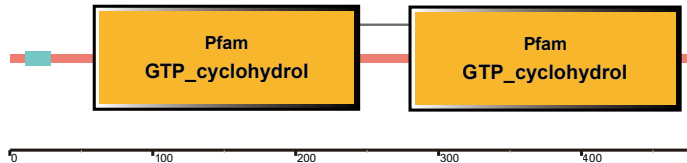

(b)

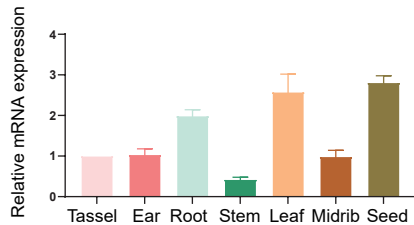

(c)

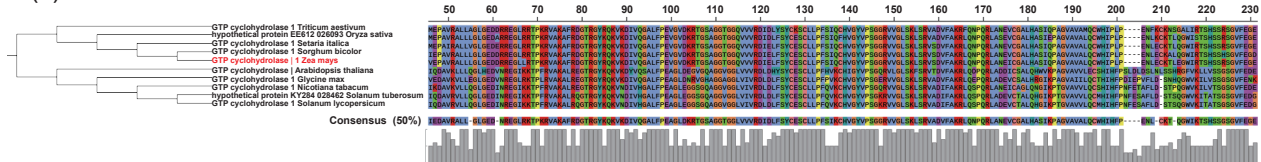

Supplement: Supplementary file 5 — Figure S5. Tissue‐specific expression and phylogenetic tree analysis. [file PBI-23-2449-s002.pdf]

(a)

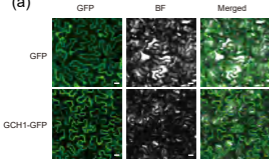

(c)

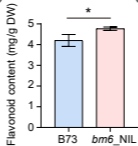

(d)

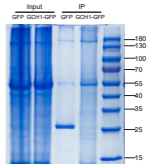

(b)

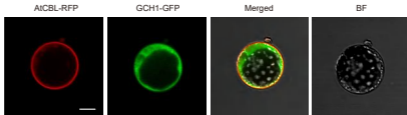

Supplement: Supplementary file 6 — Figure S6. Subcellular localization and IP‐MS analysis. [file PBI-23-2449-s004.pdf]

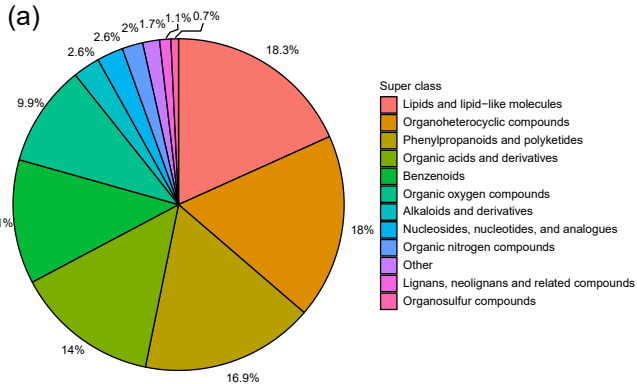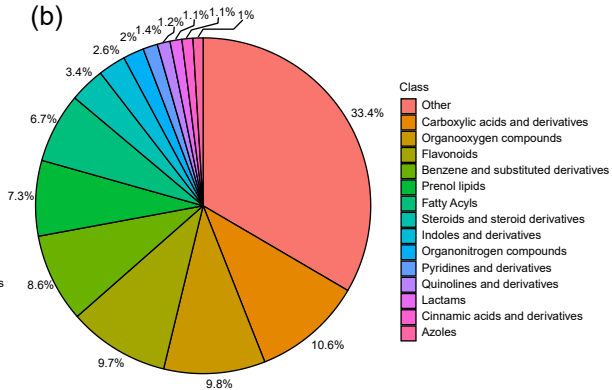

Supplement: Supplementary file 7 — Figure S7. Metabolite classification based on ClassyFire database. [file PBI-23-2449-s010.pdf]

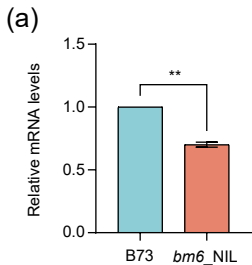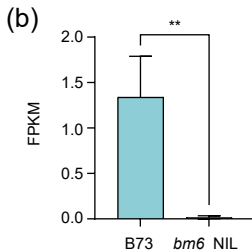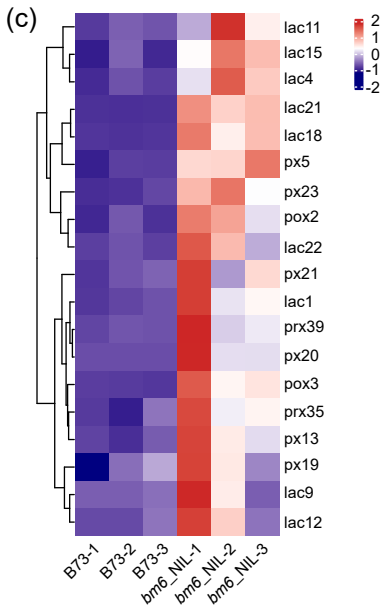

Supplement: Supplementary file 8 — Figure S8. Transcriptome and qRT‐PCR analysis. [file PBI-23-2449-s008.pdf]

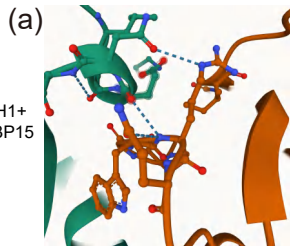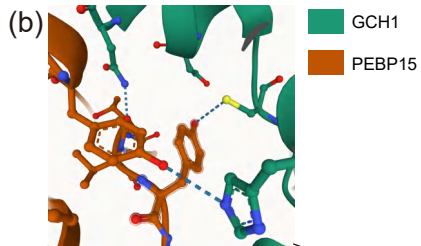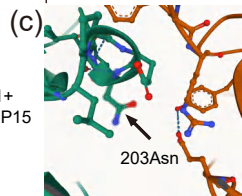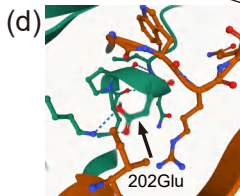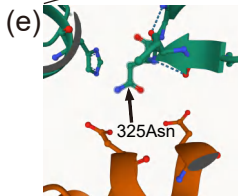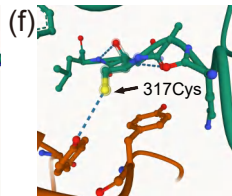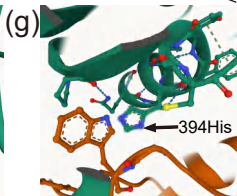

Supplement: Supplementary file 9 — Figure S9. Predicted interaction between ZmGCH1 protein and ZmPEBP15 protein using AlphaFold Server. [file PBI-23-2449-s013.pdf]

(a)

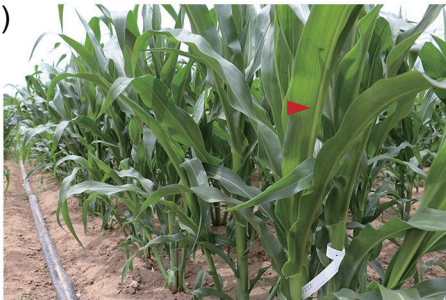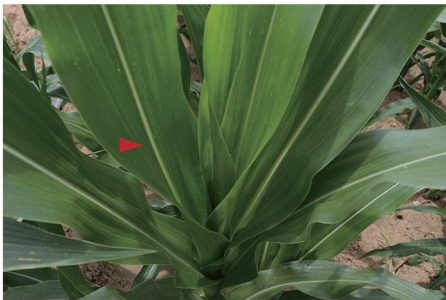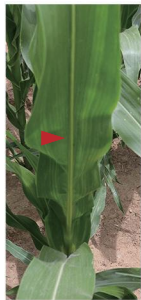

(b)

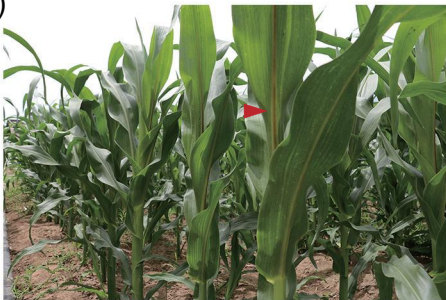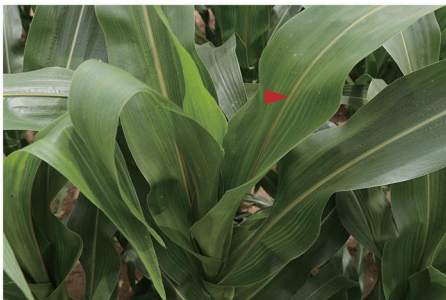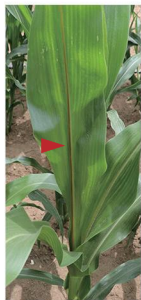

Supplement: Supplementary file 10 — Figure S10. Field phenotypes. [file PBI-23-2449-s011.pdf]

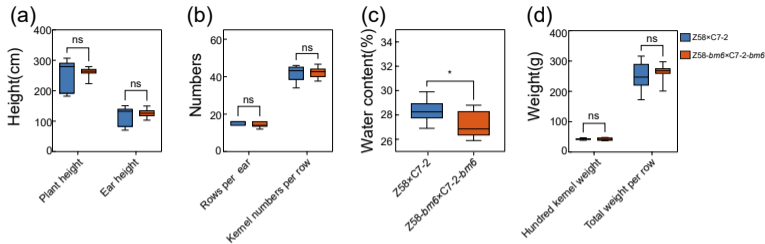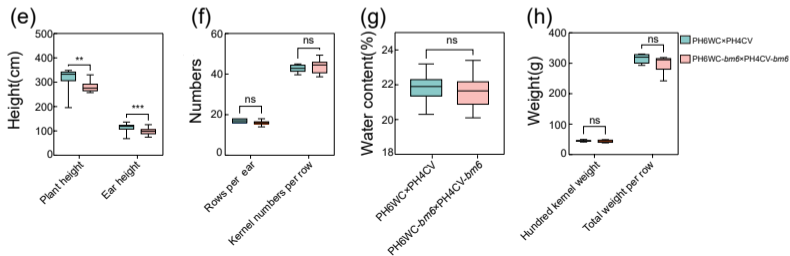

Supplement: Supplementary file 11 — Figure S11. The introduction of the bm6 gene resulted in changes to the agronomic traits of Zhengdan 958 and Xianyu 335 in Suihua city. [file PBI-23-2449-s012.pdf]

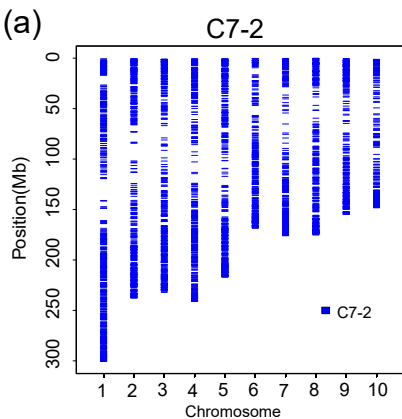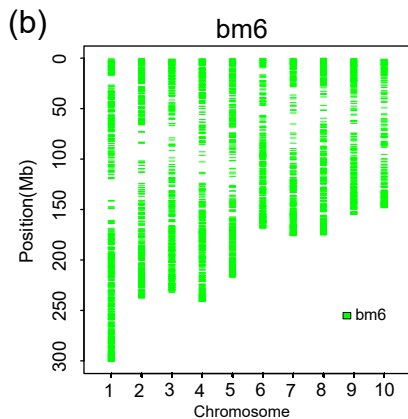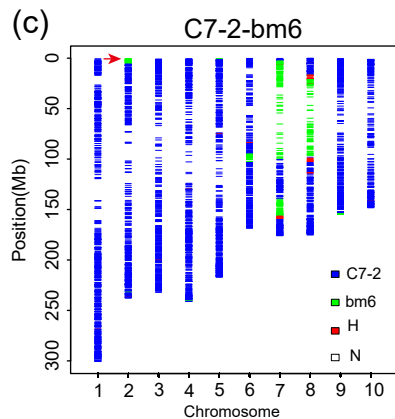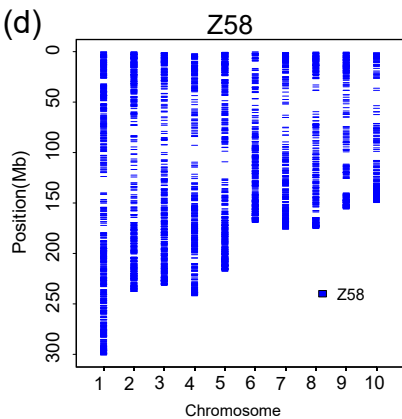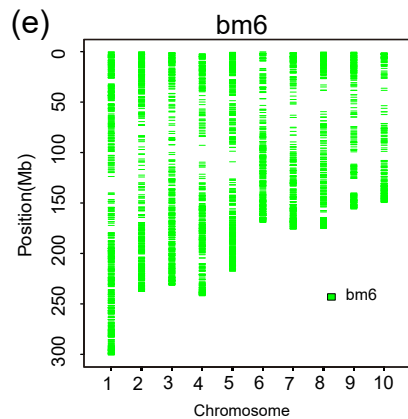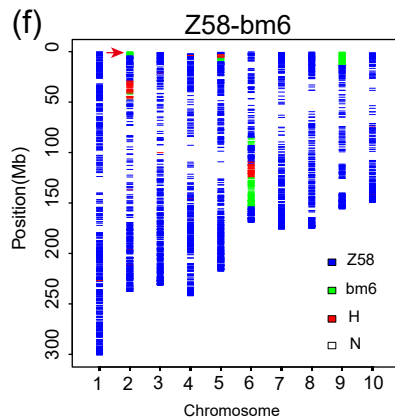

Supplement: Supplementary file 12 — Figure S12. Analysis of the parental background recovery rate in Zhengdan 958 following the introduction of the bm6 Gene. [file PBI-23-2449-s003.pdf]
